# Empowering mothers: Advancing maternal health literacy and numeracy through the introduction of Maternal and Child Health Calendar

**DOI:** 10.1177/17455057241291725

**Published:** 2024-11-20

**Authors:** Salima Meherali, Brett Matthews, David Myhre, Saba Nisa, Sobia Idrees, Ashiq Faraz, Kaleem Ullah, Roheena Shah, Zohra Lassi

**Affiliations:** 1 College of Health Sciences, Faculty of Nursing, Edmonton Clinic Health Academy, University of Alberta, Edmonton, AB, Canada; 2 My Oral Village, Inc., Toronto, ON, Canada; 3 Aga Khan Rural Support Programme, Skardu, Gilgit-Baltistan, Pakistan; 4 Robinson Research Institute, Adelaide Medical School, University of Adelaide, Adelaide, SA, Australia

**Keywords:** health literacy, numeracy, maternal, child, woman, literacy

## Abstract

**Background:**

The health literacy and numeracy skills of women in Pakistan are very low compared to other low- and middle-income countries.

**Objective:**

The aim of this study was to improve the health literacy and numeracy skills of unschooled women in Northern Pakistan by developing a Maternal and Child Health Calendar (MCHC). The MCHC utilizes locally contextualized icons to promote and enhance service utilization and maternal and child health (MCH) outcomes.

**Methods:**

We conducted a qualitative exploratory study design to understand the experiences and usefulness of the MCHC among women. We recruited the participants using purposive sampling. Using a semi-structured interview guide, we conducted individual interviews with nine Key informants, that is, Agha Khan Rural Support Staff and Community-based savings group staff and five focus group discussions with unschooled women. We followed Braun and Clarke’s steps to conduct an inductive thematic data analysis.

**Results:**

The findings of our study are categorized into the following themes: (1) the benefits of using MCHC, (2) the usefulness of the MCHC in women’s healthcare decision-making, (3) empowerment of poorly schooled women, (4) enabling numeracy and record-keeping skills, (5) MCHC implementation challenges, and (6) participants suggestions to improve the MCHC. Our findings revealed that the MCHC improved the health literacy and numeracy of illiterate or less educated women by using localized images to help them comprehend their own and their children’s health. Additionally, it effectively empowered these women in their healthcare decision-making, such as discussing family planning with their husbands. Women also suggested modifying some images in the MCHC to enhance their clarity and usefulness.

**Conclusion:**

The MCHC has the potential to safely and sustainably build basic MCH literacy and numeracy skills among both literate and illiterate women in Northern Pakistan. Further research is needed to assess its potential as a stand-alone intervention to improve MCH outcomes.

## Plain language summary

Empowering mothers: Advancing maternal health literacy and numeracy through the introduction of Maternal and Child Health Calendar

This study aimed to improve health literacy and numeracy skills among unschooled women in Northern Pakistan by developing a Maternal and Child Health Calendar (MCHC). The MCHC uses locally relevant images to help women better understand and use health services for themselves and their children. We found that it helped women in Northern Pakistan understand health information through familiar images, make informed healthcare decisions, and manage their health records. Despite some challenges and suggestions for improvement, the MCHC shows promise in enhancing health literacy and numeracy among both literate and illiterate women. Further research is needed to see if it leads to better health outcomes for mothers and children.

## Introduction

According to the World Bank,^1,2^ Pakistan is home to approximately 220 million people. In 2019, the literacy rate in Pakistan stood at 58%, with a noticeable gender gap.^3^ Females had a lower literacy rate of 44% compared to males at 69%,^3^ with rural women and girls facing even lower rates. Literature also indicates the existence of this gender gap by highlighting that Pakistan ranks poorly on the Gender Inequality Index and is 86th of 108 countries on the Social Institutions and Gender Index, a measure of discriminatory gender norms.^4,5^ Furthermore, women living in Northern/rural areas of Pakistan have limited access to formal schooling, resulting in a lack of literacy and numeracy skills.^6^ Lack of formal literacy also impacts health literacy and numeracy skills.^7^

Health literacy is defined as “the degree to which individuals have the capacity to obtain, process, and understand basic health information and services needed to make appropriate health decisions.”^8(p. 1)^ Levy et al.^9^ defined health numeracy as “the degree to which individuals have the capacity to access, process, interpret, communicate, and act on numerical, quantitative, graphical, biostatistical, and probabilistic health information needed to make effective health decisions” (p. 375). Health literacy and numeracy are closely related; for example, women with low health numeracy skills cannot read and understand basic medical directions containing numerical information, such as instructions on medication bottles or the date and time of medical appointments and are unable to calculate medical fees.^10,11^ Moreover, individuals with low health literacy and numeracy are less likely to understand health risks or to comply with medication regimes, are less familiar with screening tests, have greater difficulty managing chronic conditions, and report worse subjective health.^10–12^

Pakistan is a country with a high maternal and infant mortality rate.^13^ Pakistan recorded a high maternal mortality rate (MMR) of 154 per 100,000 live births in 2020.^14^ Moreover, rural areas face a 26% higher MMR than urban areas.^15,16^ Literature underscores the critical role of health literacy and numeracy for women in maintaining their own and their children’s health. For instance, women with lower levels of education and health literacy are less likely to seek prenatal care.^17,18^ Furthermore, with the majority of the population in Pakistan living in abject household poverty,^19^ most women are unable to afford health care from a skilled birth attendant. As a result, most deliveries occur at home, with the aid of the village midwife, older woman relative, or neighbor. This unsafe practice and behavior have had serious implications for the health of mothers and their newborns in the province.^17,18^

Gilgit Baltistan and Chitral (GBC), one of Pakistan’s five provinces, is situated at the geographically and strategically sensitive border area bounded by China, India, and Afghanistan. It comprises 10 administrative districts and is home to approximately 1.8 million people.^20^ As with the rest of Pakistan, GBC presents a complex diversity in how women and men experience inequality and disparities in education, albeit barriers to equality and empowerment are generally far greater for women than for men.^17,18^ Women in these remote areas face challenges with health literacy, numeracy, and limited access to maternal and child health (MCH) services due to financial constraints.^17,18^

To empower women, Agha Khan Rural Support staff (AKRSP) initiated a community-based savings group (CBSG) program. CBSG program serves as a vehicle to promote MCH by helping participating women of reproductive age (15–49 years) to build up the financial resources they can use to utilize MCH services. It is intended to overcome the serious challenges women face in accessing appropriate health care in time due to lack of public transport, high fares, distant health facilities, travel time, and uncertain availability of trained healthcare providers from formal healthcare sectors in rural, remote areas of GBC.

Although organizations like AKRSP have been making efforts to enhance MCH services for women and provide economic support through CBSGs,^21,22^ no initiative has been taken to promote health literacy and numeracy among illiterate women. Without health literacy and numeracy skills, the women’s efforts to securely manage their healthcare decisions (i.e., MCH) are constrained. In addition, due to low health literacy and numeracy skills, these poorly schooled women are not able to interact with the formal healthcare system. For example, they are unable to follow dosing and medical instructions, monitor the weight and height of their children, and keep up with vaccination schedules, making it more difficult for them to improve MCH outcomes.^11^ Therefore, we aimed to enhance the health literacy and numeracy skills of illiterate and innumerate women participating in GBSGs through the collaboration of the University of Alberta’s Faculty of Nursing, AKRSP, and Canadian non-profit organization My Oral Village (MOVE; https://myoralvillage.org)^23^ we developed an innovative Oral Information Management (OIM) tool, that is, “Maternal and Child Health Calendar” (MCHC).

OIM tools enable illiterate and innumerate adults to understand the information without the written text. These tools may include, but are not limited to, the use of localized, contextually based pictures, icons, and stories. Based on these principles, MCHC was designed to enhance health literacy and numeracy among illiterate women to improve MCH.

**Figure 1.**
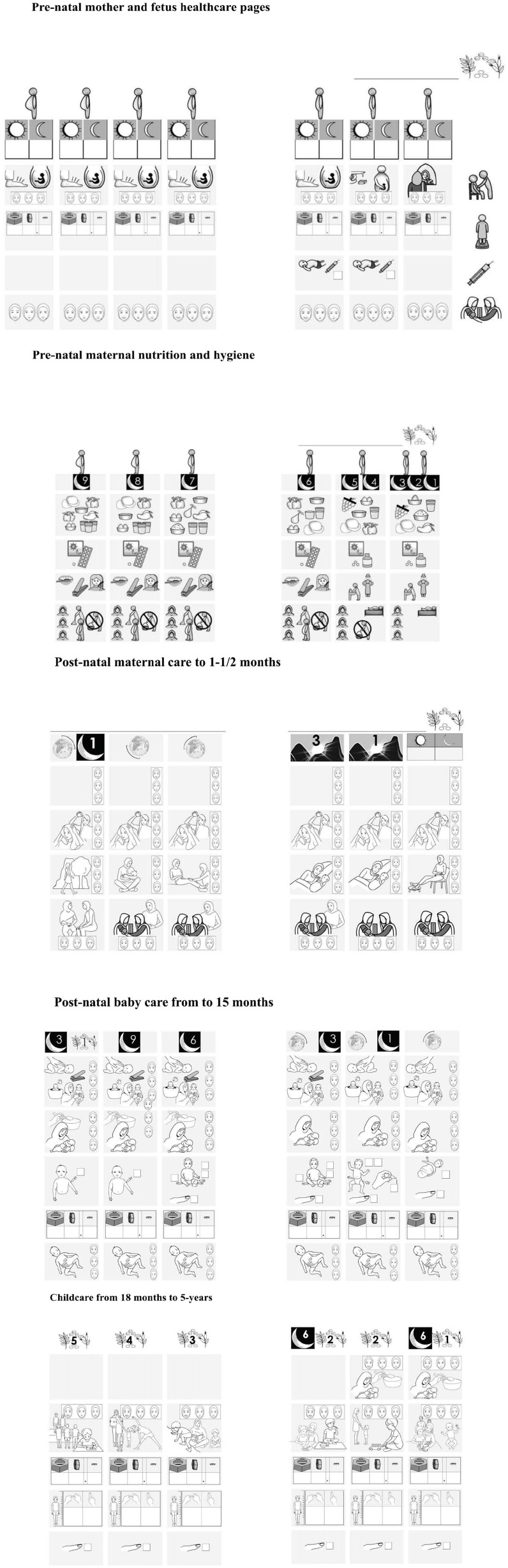
Main components of the Maternal and Child Health Calendar.

The MCHC addresses topics such as vaccinations, health clinic visits, nutrition advice, and appropriate measures for expectant mothers. The goal was to provide support and self-monitoring activities for mothers throughout the prenatal and postnatal period, and for children under 5 years. We used local references to concretize these numerical health abstractions to make them more memorable (Figure 1). This study aimed to assess the effectiveness of the MCHC and explore the experiences of AKRSP staff who conducted training sessions for illiterate women participating in CBSGs to use the MCHC calendar. We also conducted focus group interviews with women in CBSGs who received training on MCHC to understand its usefulness and usability.

## Methods

To create the MCHC, an OIM design team was formed, which included participants from AKRSP, the University of Alberta, and MOVE. This team met online for several months, with AKRSP staff often traveling to the field to meet women participants to diagnose their numeracy and record-keeping skills, test the types of icons they could quickly and easily understand, and plan how to lay out the MCHC and CBGS record-keeping booklets for ease of navigation and understanding.

### Research design

A qualitative exploratory study design was used to understand the experiences and perspectives of AKRSP staff and CBSGs women regarding MCHC. Exploratory research involves thoroughly examining human behavior, experiences, attitudes, intentions, and motivations through observation and interpretation. This method aims to understand the underlying thought processes and emotions of individuals, providing insights into how people think and feel about various phenomena.^24^ This approach ensures that the experiences and perspectives of AKRSP staff and CBSGs women regarding MCHC are thoroughly explored and understood within their specific context. Ethical approval for this study was granted by the University of Alberta Ethics Review Board (ERB #Pro00119361).

### Data collection

We recruited participants via telephone and face-to-face meetings. We used Purposive sampling to recruit participants. Purposive sampling was chosen to understand participants’ experiences involved in the pilot testing project to implement the MCHC. This included participants who either provided or received training on the MCHC to better understand the challenges and benefits of the implementation of MCHC. The focus group discussions (FGDs) were conducted in a local setting, a place where women gather for CBSGs meetings. Individual Key individual informants (KIIs) interviews were conducted online via Zoom.

A semi-structured interview guide was crafted in collaboration with a team of qualitative research experts to ensure comprehensiveness and relevance. A total of nine in-depth individual interviews were conducted with KIIs. KIIs were AKRSP staff and the managers of CBSGs who played significant roles in the development and implementation of MCHC training. Additionally, five FGDs with illiterate women from the following five districts: Mehdiabad, Ghasing, Akhonpa, Pandah, and Kharmang were conducted. Each FGD consisted of six women participants. The individual interviews and FGDs were conducted by SN, SI, AF, and RS. We utilized the Consolidated Criteria for Reporting Qualitative Research (COREQ) guidelines to ensure the rigor and transparency of this study.^25^

These interviews were conducted in Urdu, and the audio was recorded, translated, and transcribed in English. The duration of individual interviews varied between 26 to 47 min, with an average interview length of 37 min. FGDs were conducted in person, in the local language [Balti], later translated into English, and recorded using a tape recorder. The duration of FGDs was 40–50 min. We stopped the interviews and participant recruitment once the data saturation was achieved and no new information emerged from the data. No participant dropped out during this study.

### Data analysis

We conducted data analysis by following inductive thematic analysis steps outlined by Braun and Clarke, which include: (i) becoming familiar with the data, (ii) generating codes, (iii) generating themes, (iv) reviewing themes, (v) defining and naming themes, and (vi) locating exemplars.^26^ Initially, we familiarized ourselves with the data by thoroughly reviewing the transcripts of in-depth youth interviews. Subsequently, two independent reviewers (SN, SI) generated initial codes, identifying recurring patterns and concepts within the transcripts. Through collaborative efforts with the first author and team, these codes were organized into broader themes, reflecting underlying patterns and meanings in the data. The identified themes underwent rigorous review and refinement through iterative discussions between the team members to ensure consistency and coherence. Each theme was assigned clear definitions, accurately capturing its essence, and exemplars were carefully selected from the transcripts to provide concrete illustrations. This systematic approach allowed us to maintain rigor and transparency throughout the analysis, ensuring that the identified themes were accurately grounded in the data. We used qualitative software NVIVO version (12) for data analysis. The field notes and reflective memos were also kept by the research assistants.

Written consent was obtained from the literate participants, and verbal consent was obtained from the illiterate participants.

To ensure the privacy and confidentiality of the participants, pseudonyms or unique identifiers were used in place of participants’ real names to anonymize their contributions. Additionally, all data were securely stored in the University of Alberta’s data repository system and accessible only to authorized personnel involved in the research. During analysis, identifying information was removed from transcripts, and only aggregated, de-identified data were used for reporting purposes. The duration of this study was from August 2022 to December 2023.

## Results

Nine KIIs, aged between 20 and 50, participated in the study. Five FGDs were conducted with CBSGs women. The majority of the women in FGDs were married and illiterate. They ranged in age between 20 and 50. The detailed demographic details of the participants are presented in Tables 1 and 2.

**Table 1.** Demographic details of Key individual informants (KIIs).

Participants’ characteristics	*n* (%)
KIIs	9
Sex	
Male	1 (11)
Female	8 (89)
Age (in years)	
20–30	6 (67)
31–40	1 (11)
41–50	2 (22)
Marital status	
Married	6 (67)
Single	3 (33)
Level of education	
Higher secondary	9 (100)
Designation	
Social mobilizers	6 (67)
Project coordinator	1 (11)
Regional project coordinator	1 (11)
Community midwife	1 (11)
Working experience (in years)	
⩽10	5 (56)
11–20	4 (44)

**Table 2.** Demographic data for CBSGs women.

Participants’ details	*n* (%)
Age (in years)	
20–30	20 (67)
31–40	9 (30)
41–50	1 (3)
Level of education	
Higher secondary	5 (17)
Secondary	4 (13)
Middle	5 (17)
Primary	2 (7)
Illiterate	14 (47)
Marital status	
Married	28 (93)
Single	2 (7)
No. of children	
0	1 (4)
1	8 (29)
2	10 (36)
3	9 (32)
Areas of residence	
Nishan Bagh Mehdiabad	3 (10)
Khar Mehdiabad	3 (10)
Monjong	2 (7)
Panda Goma	4 (13)
Ghasing Payeen	3 (10)
Ghasing Bala	3 (10)
Akhonpa	6 (20)
Kamango Gons	3 (10)
Kamango Yul	3 (10)

CBSG: community-based savings group.

The themes that emerged from the collected data are as follows: (1) the benefits of using MCHC, (2) the usefulness of MCHC in their healthcare decision-making, (3) empowerment of poorly schooled women, (4) MCHC implementation challenges, and (5) participants suggestions for improving the MCHC.

### The benefits of using MCHC

The findings of our study represent the several benefits of using MCHC by women, such as gaining knowledge about their health and their children’s well-being, understanding pregnancy-related complications, and being able to comprehend information without formal education. The MCHC empowered women to take a more proactive role in monitoring their health during pregnancy rather than relying solely on healthcare workers who may not provide comprehensive information. Before the introduction of the calendar, women were dependent on lady health workers (LHWs) or community midwives (CMWs) in their area for women and their children’s health needs. However, they received limited information about pregnancy complications from LHWs and CMWs, as one of the CBSGs women shared that,

“I had received some information from the CMW about taking folic acid tablets, but I lacked awareness of potential complications during and after pregnancy. This calendar helped me to understand the importance of checking blood pressure and weight to avoid pregnancy-related complications” (FGDs, 2).

Participants expressed excitement and appreciation for the knowledge gained through the MCHC. Women in the community reported a lack of access to sufficient information to enhance their and their children’s health. The MCHC addressed this knowledge gap by providing comprehensive education on crucial health topics and empowering women in the community. As one of the women in FGDs shared that:

“I gained much knowledge from the MCHC training regarding maternal and child health care. It was surprising to realize that even in this advanced era, we had very little information about it, including important topics like weight management, blood tests, diet, and the significance of folic acid. Through the MCHC program, we learned about all these aspects” (FGDs, 1).

Furthermore, women shared that the calendar was easy to understand. The inclusion of icons, illustrations, and images in the MCHC significantly aided women with low or no literacy, enabling them to grasp crucial information concerning their children’s health. By using visual aids, the user-friendly design of the MCHC made it an accessible tool to a broader range of women, regardless of their educational background. One participant reported, “I like the diet, year, and months’ section in MCHC, as it is easy to understand by the icons. It is easy to remember” (FGDs, 4). Moreover, KIIs also supported this statement, as one of the KIIs expressed:

“This MCHC works best in the context where a mother has no clue how to take care of her health and her children” (P-05).

Similarly, another woman who was illiterate added that icons helped her in learning dates and schedules for vaccination and antenatal visits throughout the MCHC:

“I learned everything through the icons without having the ability to read and write. I see an icon of a woman putting her hand on her belly that shows me I have to check the movements of my baby by putting my hand on my belly during my pregnancy period. I looked at the icon of injection and learned that the vaccine is due on that particular day” (P-07).

KIIs shared their observations about women during the training of MCHC, noting that women displayed enthusiasm for the MCHC training, demonstrating a strong motivation to delve into new information. This enthusiasm was not limited to passive participation; the women actively engaged with the material and showed an interest in understanding the content. They were willing to learn this knowledge comprehensively and disseminate it among their family, friends, and neighbors. This behavior indicates a ripple effect, where the training can extend beyond the immediate participants to the broader community, potentially fostering a more informed, health-conscious community. As one of the KIIs mentioned:

“Initially, women found it challenging to be willing to get this (MCHC) training, but as they started learning, they were eager to learn more and more; even some of the women asked if they could involve their other family members like mother-in-law, sister, neighbors in the training” (P-04).

The findings indicate that introducing the MCHC positively influenced the participants’ understanding and awareness of maternal and child health care. It underscored the significance of timely interventions and a holistic approach to overall health and well-being.

### Usefulness of MCHC in their healthcare decision-making

This theme represents how women find MCHC useful in shaping their healthcare decisions, such as blood tests, weight monitoring, and post-delivery hygiene instructions. According to them, these elements were particularly vital for ensuring their own health and the well-being of their children. The ability to monitor and understand these health metrics empowers women to take proactive steps in maintaining their children’s health, breaking away from traditional practices that may have been detrimental. As one participant during FGDs said,

“I used to avoid checking my babies’ weight in their early years because of superstitions and myths. But now, I understand the importance of monitoring my child’s growth and development, now I know the importance of monitoring weight so I will definitely get it done” (FGDs, 2).

With the support of MCHC, women were able to make informed choices about their own health, as well as their families health. This ability is evident in their newfound confidence to openly discuss and manage their pregnancies, which contrasts with past practices of secrecy and resulting isolation. The MCHC has equipped them with the knowledge and tools to advocate for their health needs and seek timely medical care. For example, during interviews, some women shared that they used to hide their pregnancies until they delivered the baby, now with the help of MCHC calendar training, women raise their voices and speak for themselves, and convince their family members to visit a health clinic for their maternity regular check-ups and not to hide their pregnancy. As shared by one participant,

“I used to keep my pregnancy a secret and only tell people after the baby was born. But now, I’ve learned how important it is to have regular check-ups during pregnancy and be aware of potential complications. Knowing the scheduled dates in MCHC for my check-ups helps me share that information with my family for timely visits to the hospital” (FGDs, 3).

Another woman highlighted her motivation to change their previous routine as she shared,

“I was used to selling chicken eggs because I did not know that I should rather feed these eggs to my young children as these are good sources of nutrition for the healthy growth of my children” (P-05).

The KIIs reported women could determine their expected date of delivery using the calculations they learned during training. This ability to predict and prepare for childbirth reflects a significant shift toward self-reliance and informed decision-making in maternal health. As evidenced by KIIs,

“I do not need to ask someone about my expected date of delivery, I know when I am due and what medications [multi-vitamins, folic acid, tetanus vaccines, and iron supplements] I have to take and when. Also, I know I have to monitor my body weight throughout the pregnancy, and if I am not gaining or less gaining, I have to see a doctor” (P-01).

Furthermore, the women shared that MCHC served as a platform to make their healthcare decisions while assessing and understanding their health, as one of the participants shared,

“With the help of the face expression icon, I will monitor my health status ‘as a healthy [happy face], for example, if I feel my baby’s movement in my belly, I will mark it as a healthy sign [happy face]. if there’s any issue with my blood profile or low levels of iron, I will mark an unhealthy [sad face]. I was able to learn that I have to avoid heavy lifting through the sketch of a cross mark on the woman carrying a heavy bucket” (FGDs, 2).

The findings from our study highlight the multifaceted impact of MCHC, not only as an educational tool but also as a practical guide for women in navigating their maternal health journey with confidence and informed decision-making.

### Empowerment of poorly schooled women

All the KIIs shared that CBSG women felt empowered after gaining knowledge and training on MCHC. They expressed that these women actively shared the importance of MCHC with their husbands and family members. To some extent, these women successfully persuaded their husbands to participate in decision-making processes regarding family planning and vaccinations for both them and their children. With the support of CBSGs, these women also gained the ability to independently make decisions concerning financial expenditures related to health visits, particularly in terms of transportation costs. This sense of empowerment is evident in the way women had communicated the importance of family planning and health to their families. A KII participant succinctly stated:

“CBSG women felt empowered to talk to their family members about the importance of family planning for their health and their family’s health” (P-02).

The training provided through MCHC also had a profound impact on women’s understanding of regular prenatal health check-ups. This knowledge helped women identify and address potential health complications early on, which can help in preventing adverse outcomes. As another woman reported:

“Through these trainings, I learned many things, but the most important learning for me is about frequent check-up visits throughout the pregnancy that will help me identify any potential complications such as anemia, high blood pressure, etc. Four of my kids died due to these issues, and I never knew what were the reasons for their deaths” (FGDs, 2).

The MCHC calendar also encouraged women to be more open about their pregnancies and seek medical care when needed, which was a cultural shift. This change in behavior highlights the calendar’s role in promoting proactive health management and advocacy among women. One of the KIIs shared,

“Women were used to hiding their pregnancies until they deliver the baby, now, with the help of MCHC calendar training, women raise their voice and speak for themselves and convince their family members to visit a health clinic for their maternity related check-ups” (P-07).

Many of the women acknowledged the significance of the MCHC, stating that it allowed them to be attentive and well-informed about their health, empowering them to make timely and appropriate health decisions. One participant specifically stated:

“I love seeing the little blood drop drawing on the calendar because it reminds me it’s time for my blood test. That test helps me know if everything is okay with my health and if my blood tests are normal” (FGDs, 3).

### Enabling numeracy and record-keeping

**Figure 2.**
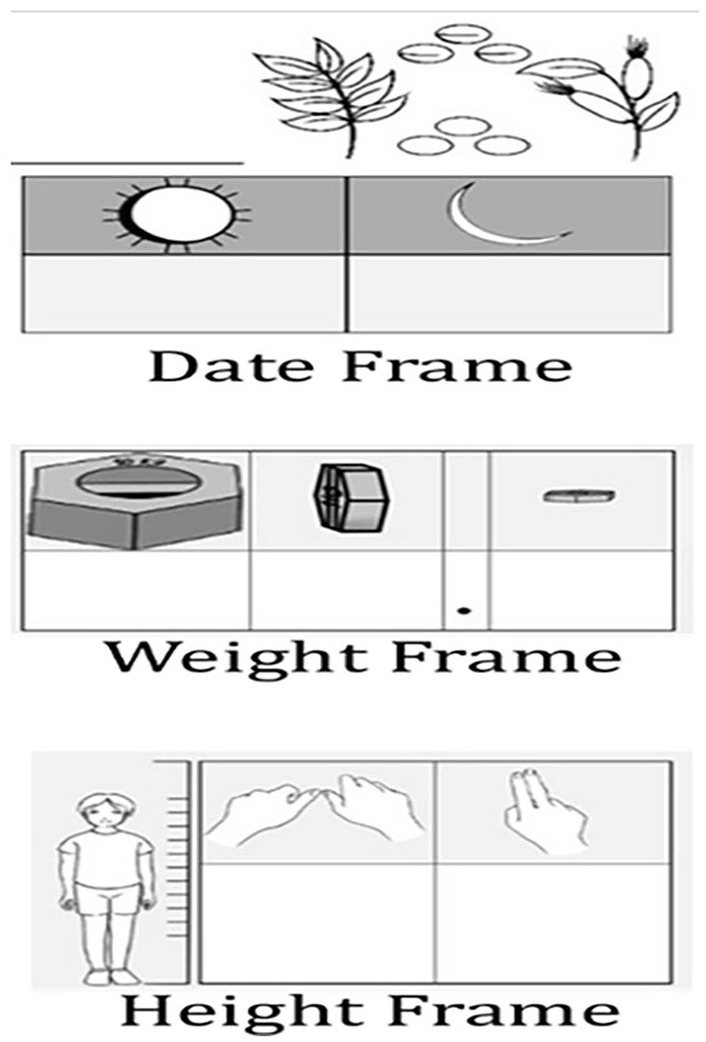
MCHC digit frame. MCHC: Maternal and Child Health Calendar.

By endline 50% of unschooled participants in this project could read a four-digit number, up from 4% at baseline 10 months earlier. And 78% could write the weight of their children in the calendar using the weight frame designed for the MCHC. The same percent of these women could find the date of their next immunization appointment on the calendar, when asked by the endline surveyor. The MCHC requires participants to read and write in three-digit frames (Figure 2). The skills required for health numeracy overlap those required for financial numeracy, beginning with digits and dates. The ability to read and write dates using an arithmetic tabular format was mainstreamed into the OIM passbooks and other records used by the CBSGs. Their appearance in the same format in the MCHC (reading from right to left) provided many women with an even better reason to learn how to use them.

Useful distinctions between the health and finance domains contributed to enhancing and deepening overall daily numeracy skills. Participants shared that tracking a child’s weight was the sole task requiring the use of decimals. Unschooled women mastered the weight frame more quickly than their schooled counterparts, who often wrote the whole number correctly, but in a single cell. The height features the use of base-12 (inches) and is supported by the graphics that informally measure inches in the villages.

These varying ways of writing digits, combined with varying reasons for writing them were presented with consistent graphical cues that signaled the purpose of each entry, so that tasks performed once were easily performed again later. As one participant commented at endline, “I was not able to write before the training. Now I can write and recognise digits easily.”

Illiterate women experienced very low HH status and were often restricted from leaving their homes for fear that they would make costly mistakes in the market or at a business place. Several reported an increase in their household status and increased freedom to move around the community due to their increased ability to read and write numbers.

### MCHC implementation challenges

This theme focuses on how participants implement sections of the MCHC in their daily lives. Participants highlighted hygiene practices as a particular concern, especially considering the challenges posed by their local weather conditions. They stated that cold weather, in particular, was a hindrance to effectively implementing hygienic measures, especially after childbirth. This indicates that environmental factors significantly impact the practical application of health guidelines provided by the MCHC. During FGDs, one woman shared her perspective,

“We find it hard to follow family planning as there’s no hospital nearby, and there are no LHWs or CMWs here, who can guide us about family planning. Also, after giving birth, it’s tough to implement hygienic measures because it’s really cold here, and taking shower after delivery is not considered good in our family” (FGDs, 4).

Participants shared concerns that one of the MCHC components is measuring blood pressure. They highlighted the challenge of implementing this component due to the absence of healthcare providers available to check their blood pressure. Furthermore, they found the family planning component to be sensitive, making it difficult for them to openly discuss family planning matters with their husbands. As one woman shared,

“It’s difficult for us to implement the family planning section because I find it difficult to discuss family planning with my husband and in-laws. It would be hard to follow the blood pressure section because there is no one to check our blood pressure” (FGDs, 1).

One of the challenges women encountered was difficulty in understanding the family planning components of MCHC. This suggests that the design of educational materials, such as the MCHC, needs to be more intuitive and culturally sensitive to ensure comprehension and usability. As shared by one of KIIs,

“Women stated that look at the icon of family planning, there is one man and a woman sitting and talking to each other, I do not understand this. If it is about family planning, it should rather have images of family planning methods such as tablets, injections, intrauterine devices, etc.” (P-05).

### Participants’ suggestions for improving the MCHC

Some participants suggested incorporating written instructions alongside the visual elements of the calendar. Women opined that the inclusion of text alongside images would enhance retention and understanding, particularly aiding them in recalling specific details that might be easily forgotten. A combination of visual and text-based information could bridge literacy gaps and ensure that the information is thoroughly understood. As one woman shared her perspective,

“If written instructions were included alongside images, it would greatly assist us in remembering the information, especially if we forget. We could rely on someone who is literate to explain the content to us” (FGDs, 5).

Women also highlighted the importance of being able to seek clarification from literate individuals in their community. Participants underscored the practical application of specific components, expressing concerns about practical skills, such as having someone demonstrate how to check blood pressure or accurately measure weight. This approach could enhance women’s abilities to implement health practices effectively. One participant articulated:

“It would be great if someone could have a practical of checking blood pressure in front of us for checking the weight. It would help us in better understanding this component” (FGDs, 1).

Another participant expressed the need for clearer instructions in specific sections of the MCHC, indicating that clarifications in these areas could facilitate better understanding and application of health guidelines. This feedback highlights the need for continuous feedback and refinement of the MCHC to meet the community’s needs, “Nutrition and vaccination section could be made clearer for better understanding” (FGDs, 2).

Moreover, KIIs discussed the necessity of ongoing training sessions for trainers to ensure its continuity in the long term. Additionally, the inclusion of an icon representing nutrition, different types of nutrients, and their importance for the health of both mother and child would be advantageous for women in understanding the significance of nutrition. A participant expressed this sentiment:

“I suggest, lady health workers should get frequent training sessions on MCHC, so that they can continue to educate CBSG women about its significance and implementation for the long term” (P-02).

Most participants highlighted the crucial need to involve and sensitize men in the future in MCHC training. They emphasized that such involvement would have widespread positive effects on improving MCH in local areas. Engaging men in the community could foster a supportive environment for women, and by extension the community to implement positive health practices. One of the KIIs participant specifically mentioned:

“Although women learned a lot about MCHC and are willing to implement new learnings to improve maternal and child health, however, women had to convince their men to take actions, for example, going for routine antenatal check-ups and getting vaccine for themselves and for their babies, so it would be much better if we also involve and sensitize men in these training projects” (P-05).

In summary, our research revealed that women expressed satisfaction with the introduction of this innovative MCHC in their area. They found it beneficial for incorporating into their daily lives, enhancing their health literacy and numeracy, and empowering them. However, some challenges in implementation were identified, and these could be addressed by providing culturally specific information.

## Discussion

Evidence suggests that maternal health literacy contributes significantly to the attainment of Sustainable Development Goals 2 (zero hunger, focusing on reducing underweight and stunting in young children) and 3 (good health and well-being, concentrating on lowering the maternal-and-child mortality ratio).^27,28^ Furthermore, enhancing maternal health literacy and numeracy has the potential to empower mothers, enabling them to make informed decisions and undertake actions that positively impact their own and their children’s well-being.^29–31^ Our research aimed to pilot test the introduction of oralized MCHC for illiterate and innumerate women in Northern Pakistan. This innovative approach draws from a similar concept of a MCH handbook, which has been successfully implemented in over 163 countries worldwide to enhance maternal, newborn, and child health.^32^ Japan was the first country to introduce the MCH handbook in 1948 to improve the health of both mothers and children.^32^ The initial MCH handbook covered the entire spectrum of pregnancy, childbirth, postpartum, and newborn care, from childhood until 6 years old. Due to Japan’s success in reducing its infant mortality rate, the MCH handbook was adapted worldwide. To date, more than 50 countries have used the integrated MCH handbook.^32^

However, many of these MCH booklets can be only used by literate women. This limitation poses a risk to illiterate women by impeding their access to crucial health information for themselves and their children. Majrooh et al.^33^ highlight the challenges illiterate women face in accessing prenatal care services in Pakistan. Sociocultural factors like low literacy level, influence of spiritual healers, poverty, and distant health facilities contribute to this inaccessibility. Our study is the first to introduce MCHC for illiterate women in Northern, Pakistan. The OIM approach contributes to empowering illiterate women, allowing them to make informed healthcare decisions and potentially improving healthcare utilization and outcomes for both mothers and children.

The findings of our study suggest that illiterate women mostly rely on healthcare providers or families for knowledge regarding their and their children’s health, especially LHWs. This finding is aligned with previous studies that reported women obtain MCH information from healthcare providers, whether at health facilities or through campaigns within their social networks (relatives, friends, or neighbors).^34–36^ However, it is important to note that merely acquiring information from healthcare providers does not automatically enhance maternal health literacy and numeracy. Although healthcare providers effectively reach the target demographic, the efficacy of their communication in delivering health messages and retention of that information is constrained by the literacy divide.

Most women in our study had little to no formal education, with the majority having no schooling at all, and only a few completed primary levels or reached a higher level. Interestingly, women showed equal interest and understanding of the MCHC regardless of their education level. In contrast to the previous researchers who found maternal health literacy linked with formal education, they found that having a difference in education level could impact the effectiveness of maternal health literacy.^35,37–40^ Uniquely, our study was the first to introduce the MCHC to both educated and less or non-educated women, utilizing localized icons/images as a communication tool for providing information about MCH. Women in our study appreciated the innovative use of icons in the MCHC, by sharing positive and beneficial remarks about the calendar.

Furthermore, in our present study, we discovered that the introduction of MCHC had a positive impact on women’s sense of empowerment. They appreciated the ability to independently learn about when to check their blood for anemia, adhere to vaccination schedules, and feel more at ease discussing family planning aspects with their husbands. As observed by AKRSPs gender advisor, several poorly schooled participants secured greater public mobility by demonstrating the ability to read and write numbers, alleviating fears within their families that they would be cheated in markets or offices. She described illiteracy as analogous in this way to a disability.

Our findings indicate that MCHC played a key role in empowering women, aligning with other research studies^41–43^ in indicating that involving mothers in developing strategies and understanding their preferred learning methods contributes to sustained empowerment. According to a systematic review, the MCH handbook improves maternal health service utilization, such as more frequent antenatal care (ANC) visits and earlier initiation of breastfeeding.^44^ It also leads to a sense of autonomy during ANC, better communication with healthcare providers, and support from family members.^45^ Our study found that women gained a better understanding of the immunization schedule for their children. The literature supports the notion that immunization coverage improves as the MCH Handbook facilitates better management of immunization records and enhances women’s understanding of vaccines such as the Hepatitis B vaccine for newborns.^46,47^ In an Indonesian study by Osaki et al.,^48^ the MCH handbook intervention significantly increased children’s immunization rates, jumping from 25.1% to 47%.

Our research revealed that the women were facing economic challenges and resided in remote areas. They expressed concerns about the limited availability of healthcare services in their communities. Therefore, it is imperative to implement some icons-based educational tools such as MCHC and training programs for illiterate women in areas where accessibility to healthcare services is limited. Social determinant variables such as residential location, educational background, and family financial status significantly influence the health outcomes of mothers and children, as emphasized in previous studies.^49–52^ Nevertheless, cultural barriers posed challenges to MCHC implementation, for example, with hygiene practices not culturally recognized as essential. Addressing social determinants impacting maternal and child healthcare in Pakistan is imperative. Immediate and decisive policy interventions are indispensable to tackle the profound social determinants impacting maternal and child healthcare in Pakistan. Prioritize initiatives that elevate the status of women through educational and empowerment programs. This high-reaching policy approach is essential to bring about comprehensive improvements in MCH outcomes.

### Strengths and limitations

This research has significant strength due to its innovative and participatory methodology, which is by actively involving women in the development of the MCHC. This approach enhances the tool’s relevance and user understanding. Second, we provided 12 weeks of training to the women, which helped them in the hands-on practice of MCHC.

However, there are certain limitations of this study; given this study’s exclusive focus on Northern Pakistan, the applicability of the findings to broader geographical regions or diverse contexts may be limited. Moreover, incorporating statistical measures could provide more strength to the qualitative findings or identified themes. Furthermore, the study identified the short-term impacts of MCHC. To strengthen the breadth of our research, it is crucial to delve into the long-term sustainability and effectiveness of the MCHC.

## Conclusion

The MCHC stands as a key player in the pursuit of equitable healthcare access and improved well-being for women and children in challenging sociocultural and geographical contexts. Sustainability and long-term impact, and the prospects for additional learning through repetitive use, will require further monitoring.

## Supplemental Material

sj-docx-1-whe-10.1177_17455057241291725 – Supplemental material for Empowering mothers: Advancing maternal health literacy and numeracy through the introduction of Maternal and Child Health Calendar Supplemental material, sj-docx-1-whe-10.1177_17455057241291725 for Empowering mothers: Advancing maternal health literacy and numeracy through the introduction of Maternal and Child Health Calendar by Salima Meherali, Brett Matthews, David Myhre, Saba Nisa, Sobia Idrees, Ashiq Faraz, Kaleem Ullah, Roheena Shah and Zohra Lassi in Women’s Health

## Declaration

**Ethics approval (include appropriate approvals or waivers) and consent to participate (include appropriate consent statements):** This study was performed in line with the principles of the Declaration of Helsinki. The survey is approved by the University of Alberta ethics review board (Pro00119361). The University of Alberta’s consent protocols were used during the study. Written consent was obtained from the literate participants, and verbal consent was obtained from the illiterate participants.

**Consent for publication (consent statement regarding publishing an individual’s data or image):** Participants provided verbal consent for sharing their data for publication and dissemination.

**Availability of data and material (data transparency):** Research data will be kept in the Health Research Data Repository at the Faculty of Nursing, University of Alberta, a secure and confidential virtual research environment used to store data through its life cycle. All data generated or analyzed during this study are included in this published article (and its Supplemental Material). Appendix/Supplemental Material are also available online (https://doi.org/10.5281/zenodo.7939868).
