# Supplementary material for: Empowering mothers: Advancing maternal health literacy and numeracy through the introduction of Maternal and Child Health Calendar
Source: Womens Health (Lond). 2024 Nov 20;20:17455057241291725. doi: 10.1177/17455057241291725 (PMC11580089; doi:10.1177/17455057241291725)
Supplement: sj-docx-1-whe-10.1177_17455057241291725 – Supplemental material for Empowering mothers: Advancing maternal health literacy and numeracy through the introduction of Maternal and Child Health Calendar [file sj-docx-1-whe-10.1177_17455057241291725.docx]

**MCHC Questions (FGDs with women in CBSGs)**

Main themes to cover (Both open and closed in ended questions) for FGDs

1. Why maternal and child health is important?
2. Have you learned anything about MCH in the past and who taught you (such as LHW, CHW, CMWs, elder in the family), how and what you have learned?
3. What is your opinion about the MCHC (show the calendar to participants)
4. Is it useful (why and how?)

Some probes:

Is it simple to use?

It provides the information important for MCH?

Do you understand the icons and images?

It meets your information needs related to MCH?

Is it fun to use?

1. What have you learned from the MCHC? Can you identify something specific that you know now but did not know before you received the calendar and were trained on it?

Probes:

Can you be more specific, please?

What was it about the weight frame/nutrition guide/ etc etc. that you

learned?

Did anyone else find that they learned about this? Was your experience

the same/different?

1. Are any of the icons particularly significant to you? Which ones? Why?
2. Which section of this calendar you think is more important? Why?
3. Which section of this calendar you think is easy to follow
4. Which section is difficult to follow? Why?
5. Did you learn anything from other members of your CBSG about the MCHC? What information did you learn from other members?
6. Did you share anything that you knew about the MCHC with other members of your CBSG? What information did you share?
7. Are you satisfied with this approach of learning?
8. Is there any section of this MCHC you would like to change/modify (what & why?)
9. Any suggestions to change this MCHC?

Tell me some positive aspects

Tell me some negative aspects

1. Will you use it in your everyday life to monitor your own and your child health and vaccination?
2. Are you going to use it in the future?
3. Would you recommend this MCHC to your friend or other family members?

**KIIS Interview Guide (CBSG 3 literate Women)**

1. Why maternal and child health is important?
2. What is your opinion about the MCHC (show the calendar to participants)
3. Is it useful for women with low literacy (why and how?)

Some probes:

Is it simple to use?

It provides the information important for MCH?

Do you understand the icons and images?

It meets the information needs of women with low literacy related to MCH?

Is it fun to use?

1. Which section of this calendar you think is more important? Why?
2. Which section of this calendar you think is easy to follow
3. Which section is difficult to follow? Why?
4. How you can support women in CBSGs to learn MCHC?
5. Do you think women will use this calendar for healthcare decision making? How?
6. Is there any section of this MCHC you would like to change/modify (what & why?)
7. Any suggestions to change this MCHC?

Tell me some positive aspects

Tell me some negative aspects

1. Will you use it in your everyday life to monitor your own and your child health and vaccination?
2. Would you recommend this MCHC to your friend or other family members?
3. What you think your role will be in the sustainability of MCHC among women in your community?

**KIIS Interview Guide (AKRSP 5-6 Staff memebers)**

1. What is your opinion about MCHC

Is it useful why and how

Do you think women with no or low literacy can easily use it to record their own and their child health matters?

1. What was your experience when you are providing training to women on MCHC?
2. What response you observed when you introduced MCHC with women in CBSGs?

Probe – Women showed interest

They were excited with this learning strategy

1. In your opinion how MCHC will help women in healthcare decision making? How
2. Do you think this is good health literacy and numeracy strategy for women with low and no literacy?
3. Do you think MCHC will empower women and increase their agency in healthcare decision making
4. Is there any section of MCHC you think need to be changed? What and why?
